# Daily step counts before, during, and after COVID-19 pandemic: a smartphone-tracking study of university students in Egypt and Saudi Arabia

**DOI:** 10.1186/s12889-023-16068-x

**Published:** 2023-07-17

**Authors:** Mohamed Aly, Mezna AlMarzooqi, Sheref Mohamed, Turki Mohsen Alzahrani, Osama Abdelkarim

**Affiliations:** 1 Faculty of Physical Education, Assiut University, Assiut, 71515 Egypt; 2 Department of Sport Sciences, College of Education, Taif University, Taif, 21944 Saudi Arabia

**Keywords:** COVID-19, Daily step counts, Physical activity, University students

## Abstract

**Background:**

COVID-19 pandemic has drastically changed people’s lifestyles and daily routines around the world. This study aimed to investigate changes in daily step counts – as an indicator of physical activity – before, during, and after the pandemic among university students in Egypt and Saudi Arabia and to identify regional and gender factors that may have influenced physical activity during these periods.

**Methods:**

A total of 1273 university students from Egypt (Assiut University and Zagazig University) and Saudi Arabia (Taif University, King Faisal University, and Imam AbdulRahman Bin Faisal University) provided valid data on their daily step counts recorded by a smartphone application (iPhone Health App) from January 2019 to December 2021. The data was divided and averaged based on three periods: the pre-pandemic year (January to December 2019), the first pandemic year (January to December 2020), and the second pandemic year (January to December 2021).

**Results:**

The results showed a significant decrease in daily steps from pre-pandemic to the first pandemic year, followed by an increase in the second pandemic year. However, daily step counts did not fully recover to pre-pandemic levels in male Egyptian and marginally in male Saudi participants. In both nationalities, female participants did not show a significant difference in daily steps between the pre-pandemic and second-pandemic years. Female Egyptian participants had significantly lower daily step counts than male Egyptian participants, and a gender difference in daily steps was also observed in Saudi female participants in the pre-pandemic and the second pandemic year but not during the first pandemic year.

**Conclusion:**

These findings outline the need for strategies in Egypt and Saudi Arabia to promote physical activity and reduce sedentary behavior, especially among young women, to mitigate the negative consequences of COVID-19 and meet physical activity guidelines.

## Background

The potential negative consciences of the COVID-19 pandemic on health outcomes have garnered significant attention and are expected to have a long-lasting effect on people’s health and lifestyle. The rate of exercise and physical activity has dramatically decreased due to social restrictions related to the pandemic measures [1, 2], which has a direct negative impact on the lifestyle of individuals among all populations, regardless of their economic and social status [3–5]. The first cases of novel coronavirus were first detected in December 2019, with the virus spreading rapidly to other countries across the world. This led WHO to declare a Public Health Emergency of International Concern and to characterize the outbreak as a pandemic on 11 March 2020. To contain the spread of the pandemic, all countries implemented extreme restrictions such as quarantines, lockdowns, closure of public and private establishments, and restriction of population mobility and gathering [4, 6]. In Saudi Arabia, the government imposed a nationwide lockdown starting on March 23, 2020, which lasted several months. Similarly, Egypt implemented a nationwide lockdown on March 25, 2020, which continued until June 27, 2020. As a result of the pandemic and the subsequent lockdowns, many educational institutions in Saudi Arabia and Egypt shifted from traditional face-to-face teaching to online teaching, followed by hybrid teaching, which combines face-to-face and online teaching. In Saudi Arabia, the academic year started on August 30, 2020, and online and hybrid teaching continued until the end of the academic year in June 2021. In Egypt, online and hybrid teaching was also implemented during the academic year 2020–2021, starting on October 17, 2020, and continued until the end of the academic year in June 2021. These measures have enormously changed the behavior of all populations (e.g., eating habits and lifestyle) to be more sedentary and physically less active [7, 8].

Globally, before the pandemic, the levels of physical inactivity were already classified as a major public health problem, with more than 25% of all adults failing to meet WHO-approved physical activity guidelines for maintaining good health [9]. In the Middle East and North Africa region (MENA), the prevalence of physical inactivity among adults, as determined by WHO daily step counts surveys, was over 40% in all Arab countries except Egypt, 32%, and it reached 68% in KSA [10]. These percentages indicate a lack of physical activity culture and high levels of sedentary behavior associated with social-cultural habits [11–14]. While the pandemic has been raging for over two years, accumulating evidence suggests that it is just the beginning and will have a long-lasting effect on our lives [15, 16]. Therefore, it is important to understand how health pandemics impact our lifestyles and health, especially in countries with high levels of physical inactivity, considering gender and cultural effects [17].

Fitbit data has shown that the pandemic has caused a significant decrease in physical activity, with more than 30 million users experiencing a decline in step counts of between 7% and 38% during the week ending March 22, 2020, compared to the same period the previous year [18]. Although studies have confirmed this decrease in daily steps as a measure of physical activity [19, 20], it remains unclear whether people have been able to restore their pre-pandemic activity levels, especially in light of gender and lifestyle differences between countries. Cultural and socioeconomic differences can also make comparisons between nations difficult. While investigations have shown some similarities in behavior change [16, 21], physical activity levels in the MENA region have been rarely studied, particularly in Egypt and Saudi Arabia.

Smartphone technology, such as mobile health apps, has been widely used to monitor daily physical activities, both currently and retrospectively [22]. These apps have been recognized as a valid and reliable methodology, especially among adults, to track health-related physical activity status during the pandemic. Mobile health apps typically use the sensors in smartphones to collect data on step counts, distance traveled, and other physical activity-related metrics. The app then uses algorithms to analyze this data and provide users with feedback on their activity levels, such as the number of steps taken and the distance covered. Thus, these apps can provide valuable information on changes in physical activity levels before and after the pandemic [22–24]. This can help to understand the impact of COVID-19 on physical activity and the associated health effects.

Given the lack of data at both the regional and country level, continuous surveillance of physical activity participation is essential to track progress toward regional and global physical activity targets, as well as to monitor the negative consequences of the COVID-19 pandemic. Therefore, the primary aim of the present study was to examine changes in daily step counts during three periods: pre-pandemic (2019), first pandemic year (2020), and second pandemic year (2021) among Egyptian and Saudi university students. The secondary aim is to examine gender and region as moderators. We hypothesized that there would be a reduction in daily step counts from the pre-pandemic to the first pandemic year but an increase in the second pandemic year. Gender and lifestyle differences between Egypt and Saudi Arabia might moderate the negative impact of COVID-19.

## Methods

### Study design

A retrospective study assessed the changes in daily step counts pre COVID-19 pandemic, first pandemic year, and second pandemic year among Egyptian and Saudi university students.

### Recruitment and participants

Participants were recruited using a convenience-based sampling approach. The sample consisted of university students from two countries: Egypt (enrolled at Assiut University, Women’s College of Physical Education, and Zagazig University) and Saudi Arabia (enrolled at Taif University, Prince Sultan Military College for Health Sciences, King Faisal University, and Imam AbdulRahman Bin Faisal University). Potential respondents were electronically invited through various social applications (e.g., WhatsApp, Messenger, Instagram). The study’s inclusion criteria were as follows: participants must be 18 years or older, currently enrolled as university students, have lived in Egypt or Saudi Arabia for at least three years, and reported using their mobile phones for most of their daily activities. Once eligibility was determined, individuals agreed to participate through an anonymous online information sheet describing the study procedures. A total of 1273 participants, with 312 male and 333 female students from Egypt and 299 male and 329 female students from Saudi Arabia were included. The study protocol was approved by the Institutional Review Board of the Faculty of Physical Education at Assiut University in Egypt and Taif University in Saudi Arabia.

### Measures

Using an online survey, a range of demographic data (i.e., sex, age) and anthropometric data (height and weight), from which we calculated body mass index (BMI), were requested from participants in the first part of the survey. Moreover, they were requested to inform their daily step counts average per month recorded by their personal smartphone from January 2019 to December 2021. Participants were also asked to rate how frequently they usually keep their smartphones with them during daily activities on a scale from one to five. A rating of one indicated that the participant did not carry their smartphone at all times, while a rating of five indicated that the smartphone was carried at all times.

### Data collection and outcomes

According to our preliminary survey, it was found that a vast majority of university students (over 95%) owned smartphones with a step counter application. Additionally, 60% of the participants reported using the Apple Health application, which is a pre-installed mobile app on iPhones that enables users to monitor and track their health and fitness data, including step count (Apple Inc., Cupertino, CA, USA). The analysis was limited to participants who reported carrying their iPhones with them during most of their daily activities. Out of the total 2513 collected records, 1699 were valid and eligible for analysis, while 814 were invalid due to incomplete data. Out of these 1699 valid records, 1273 were from the Apple Health application, and 426 were from Android devices using various applications. For research purposes, only the data obtained from the Apple Health app was used. To determine the average daily step count, the data was analyzed for three periods: pre-pandemic year (January 2019 to December 2019), first pandemic year (January 2020 to December 2020), and second pandemic year (January 2021 to December 2021).

### Procedures

An online survey in Arabic was conducted using Microsoft Forms between January 15 and April 15, 2022. Participants were screened online to establish eligibility for the study, and those who met the criteria were asked to report their sex, height, weight, and average daily step count for each month from January 2019 to December 2021. The survey took approximately 10 min to complete, and no incentives were offered to participants.

### Data analysis

Data were screened for univariate outliers using standardized scores (*z* > ± 3.29) and multivariate outliers using the Mahalanobis distance test (*p* < 0.001) [25]. The parametric assumptions that underlie ANOVA mixed model were examined. Averaged daily step counts were analyzed by 3 (Time: pre-pandemic, first pandemic year, and second pandemic year) × 2 (Gender: male and female) × 2 (Region: Egyptian and Saudi) ANOVA mixed model. Analysis was subjected to a Greenhouse–Geisser adjustment when the assumption of sphericity was violated. Main and interaction effects were followed by post hoc tests and multiple comparisons of t-tests with Bonferroni corrections. The effect size was expressed as a partial eta-squared (η^2^) to determine the magnitude of the effect when a significant main and interaction effect was reached. An alpha level of 0.05 was set for statistical significance. The statistical analyses were conducted using SPSS 25.0 (IBM Inc., Chicago, IL, USA).

## Results

Table 1 presents the participants’ demographics (i.e., age, weight, height, and BMI) and daily steps across the three periods: pre-pandemic (2019), first pandemic year (2020), and second pandemic year (2021).

**Table 1:** Participant characteristics and averaged daily step counts

Variables	Egyptian (*n* = 645)						Saudi (*n* = 628)					
	male (*n* = 312)		Female (*n* = 333)		Total		male (*n* = 299)		Female (*n* = 329)		Total	
	Mean	SD	Mean	SD	Mean	SD	Mean	SD	Mean	SD	Mean	SD
Age (years)	20.66	4.53	19.59	2.73	20.109	3.747	20.07	3.34	20.64	2.39	20.367	2.892
Weight (kg)	1.75	0.06	1.63	0.05	1.687	0.083	1.71	0.07	1.59	0.09	1.650	0.099
Hight (m)	68.69	9.72	60.90	8.55	64.669	9.922	72.97	16.06	61.94	14.68	67.190	16.298
BMI (kg/m^2^)	22.45	3.48	23.07	3.43	22.769	3.469	25.01	5.96	24.64	6.44	24.812	6.216
Averaged daily steps												
Pre-pandemic	4622.37	1935.82	4298.97	1762.07	4455.400	1853.782	3659.08	1730.66	3916.01	1729.21	3793.680	1733.281
First pandemic year	3732.45	2117.15	3340.89	1702.78	3530.300	1922.940	2880.54	1703.59	2866.82	1489.45	2873.360	1593.725
Second pandemic year	4641.77	2268.81	3946.23	1807.36	4282.680	2071.428	3457.52	1894.69	3725.81	1927.89	3598.070	1915.334

The ANOVA revealed that there was a main effect of Time, *F* (1.92, 2441.69) = 160.76, *p* < 0.01, *η* ^2^ = 0.112, with a lower number of daily steps in the first pandemic year followed by the second pandemic year, and then pre-pandemic year. There was also a main effect of Gender, *F* (1, 1269) = 106.21, *p* < 0.01, *η* ^2^ = 0.077, with a lower number of daily steps in females than males. There was also a main effect of Region, *F* (1, 1269) = 66.43, *p* < 0.01, *η* ^2^ = 0.020, with a lower number of daily steps in Saudi than in Egyptian.

**Fig. 1:**
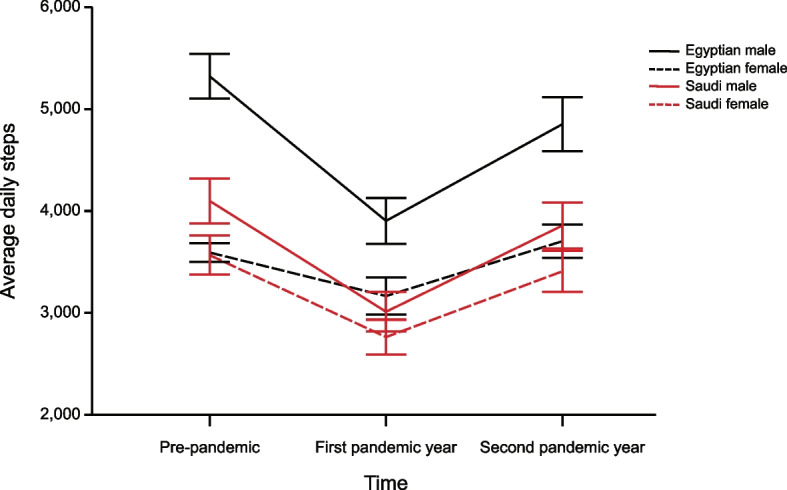
Interaction plot of time, gender, and region for average daily steps. Error bars represent 95% CI

The effects of the interactions of Time × Gender, *F* (1.92, 2441.69) = 16.76, *p* < 0.01, η_p_^2^ = 0.013, Gender × Region, *F* (1, 1269) = 25.75, *p* < 0.01, *η* ^2^ = 0.020, and Time × Gender × Region, *F* (1.92, 2441.69) = 5.45, *p* < 0.01, *η* ^2^ = 0.004, were also significant (see Fig. 1).

Post hoc analyses comparing the Time differences within each region and gender revealed that daily step counts did not fully recover to pre-pandemic levels in male Egyptian (*p* < 0.01) and marginally in male Saudi participants (*p* = 0.05). Female participants did not show a significant difference in daily step counts between the pre-pandemic year and the second pandemic year in both regions (*p* > 0.18).

Analyses comparing the Gender difference within each period of time and region revealed that the Egyptian female participants had significantly lower daily step counts than the male participants (*p* < 0.01), whereas lower daily steps were observed for the Saudi female participants relative to the male participants in pre and second pandemic year (*p* < 0.01), but not during the first pandemic year (*p* = 0.08).

Secondary analyses comparing the regional differences within each period of time and gender revealed that the Egyptian male participants had significantly higher daily step counts than Saudi male participants across all three periods (*p* < 0.01), whereas daily step counts did not significantly differ between Egyptian and Saudi female participants in pre-pandemic and during the second pandemic year (*p* > 0.05). However, during the first pandemic year, daily steps were higher in the Egyptian female compared to Saudi female participants (*p* < 0.01).

## Discussion

We examined changes in university students’ physical activity during the COVID-19 pandemic according to three periods: pre-pandemic, first pandemic year, and second pandemic year, using daily step counts through a smartphone application. To the best of our knowledge, this is the first study to report the daily step counts of Egyptian and Saudi university students across an extended period of time. The findings revealed that daily step counts were influenced by gender, as well as potential cultural differences between Egypt and Saudi Arabia. Additionally, our findings demonstrated variations in daily step recovery in the second pandemic year. Moreover, our data showed insufficient physical activity was prevalent in both Egyptian and Saudi university students, particularly among females.

Our data showed a significant reduction in daily step counts during the first pandemic year, followed by an increase in the second pandemic year. This finding suggests that the pandemic had a negative impact on physical activity levels among university students, which is consistent with previous studies conducted among general populations around the world during the COVID-19 pandemic [17, 23, 26–35]. This indicates that the pandemic preventive measures have completely changed the university students’ lifestyle, especially for teaching activities that have been turned to be delivered in digital form with extreme restrictions related to any campus activity [26–30]. The reduction of time spent on daily step counts could be explained by the fact that students get used to the less active lifestyle (e.g., online teaching) during the first pandemic year. Our study also suggests that university students have gradually begun to restore their daily activities as before COVID-19, which could be due to the gradual lifting of restrictions and the re-establishment of daily routines. Additionally, our data showed that females had lower daily steps than males across all three periods. This finding is also consistent with previous research on gender differences in physical activity levels, which has shown that males are generally more physically active than females [36–39].

The present study suggests that there is interaction between time, gender, and region on daily step counts. This implies that the impact of the pandemic on daily steps varied based on these three factors. Specifically, analyses examining the time differences within each region and gender revealed that daily step counts did not fully recover to pre-pandemic levels in male Egyptian and marginally in male Saudi participants. This suggests that male students in both countries may have been more significantly impacted by the pandemic on their physical activity levels compared to female students. However, it is important to consider that female students may have had lower baseline activity levels, which could explain why they appeared to recover faster than male students. Furthermore, the analyses comparing gender differences within each period of time and region revealed that the Egyptian female participants had significantly lower daily step counts than the male participants, while lower daily steps were observed for the Saudi female participants relative to the male participants in pre and second pandemic year, but not during the first pandemic year. This suggests that the gender differences in daily step counts varied by region and over time during the pandemic. Thus, these findings highlight the importance of considering gender and the cultural and social context when examining the impact of the pandemic on physical activity levels. Specifically, gender, regional differences in social norms, cultural values, and daily routines may play a role in determining the negative circumstances of COVID-19. For instance, in Saudi Arabia and Egypt, cultural norms may have made it more difficult for women to engage in physical activity in general, which could explain the greater reduction in daily step counts among female students [40–43]. Strategies to promote physical activity should take these factors into consideration to ensure that interventions are tailored to meet the specific needs of different populations.

Daily step counts of university students in Egypt and Saudi Arabia were significantly lower even before the onset of the COVID-19 pandemic compared to the recommended guidelines (e.g., 10,000 steps/day) and individuals in other countries [44–47]. A meta-analysis highlighted the prevalence of inactivity among adults and youth in the MENA region [13]. The study found that 25.6% of youth were sufficiently active. With limited data available on physical activity behaviors in the MENA region, the current study provides updated data regarding the current situation among university students in Egypt and Saudi Arabia. This information can assist policymakers in developing targeted interventions to promote physical activity and improve overall health outcomes.

The presented results should be interpreted in light of the following limitations. First, the sample was collected using a convenient sampling approach and only included university students from Egypt and Saudi Arabia, which may limit the generalizability of the findings to other populations. Second, the study relied on daily step counts as a measure of physical activity, which may not capture other forms of other physical activity types and intensities. However, daily steps are a commonly used indicator for physical activity levels, especially in studies conducted in developing countries where resources for measuring physical activity are limited. Third, the study relied on self-reported data from a smartphone application, which may exclude individuals who do not use mobile health applications, potentially biasing the sample. However, this issue might have had a limited impact on our study findings due to the relatively large sample size that we used. By including a substantial number of participants in our study, we aimed to increase the representativeness of our findings and reduce the potential impact of this limitation. Fourth, the cutoff date used in the current study did not consider the lockdown period and government restrictions. The choice of a cutoff date in our study was made based on the aim of our study, which primarily focused on changes in daily steps during the pandemic rather than the exact date of the lockdown. Therefore, the results of this study should be interpreted with caution, and further research is needed to confirm these findings and address these limitations.

## Conclusion

In conclusion, the COVID-19 pandemic had a significant impact on the physical activity levels of university students in Egypt and Saudi Arabia, as indicated by their daily step counts. The results of this study suggest that there was a decrease in daily step counts during the first year of the pandemic, followed by a partial recovery in the second year. However, the recovery was not uniform, as male Egyptian and Saudi participants did not fully return to pre-pandemic activity levels. Moreover, there were regional and gender differences in the physical activity levels of the participants, with female Egyptian participants having lower daily step counts than male Egyptian participants, and a gender difference in daily steps was also observed in Saudi participants. These findings highlight the importance of promoting physical activity among university students and the need for targeted interventions to address the disparities observed in physical activity levels.

## Data Availability

The datasets used and/or analyzed during the current study are available from the corresponding author on reasonable request.
